# Lignin Degradation and Its Use in Signaling Development by the Coprophilous Ascomycete *Podospora anserina*

**DOI:** 10.3390/jof6040278

**Published:** 2020-11-11

**Authors:** Moussa Dicko, Roselyne Ferrari, Narumon Tangthirasunun, Valérie Gautier, Christophe Lalanne, Farida Lamari, Philippe Silar

**Affiliations:** 1 Université Sorbonne Paris Nord, CNRS LSPM UPR 3407, 93430 Villetaneuse, France; moussa.dicko@lspm.cnrs.fr (M.D.); farida.lamari@univ-paris13.fr (F.L.); 2 Université de Paris, Laboratoire Interdisciplinaire des Energies de Demain (LIED), F-75006 Paris, France; roselyne.ferrari@univ-paris-diderot.fr (R.F.); tonamsomka@gmail.com (N.T.); valerie.gautier@univ-paris-diderot.fr (V.G.); ch.lalanne@mac.com (C.L.)

**Keywords:** biomass, fungal degradation mechanisms, *Podospora anserina*, peroxide, pyrolysis-GCMS, miscanthus

## Abstract

The filamentous fungus *Podospora anserina* is a good model to study the breakdown of lignocellulose, owing to its ease of culture and genetical analysis. Here, we show that the fungus is able to use a wide range of lignocellulosic materials as food sources. Using color assays, spectroscopy and pyrolysis–gas chromatography mass spectrometry, we confirm that this ascomycete is able to degrade lignin, primarily by hydrolyzing β–O-4 linkages, which facilitates its nutrient uptake. We show that the limited weight loss that is promoted when attacking *Miscanthus giganteus* is due to a developmental blockage rather than an inefficiency of its enzymes. Finally, we show that lignin, and, more generally, phenolics, including degradation products of lignin, greatly stimulate the growth and fertility of the fungus in liquid cultures. Analyses of the *CAT^ΔΔΔΔΔ^* mutant lacking all its catalases, pro-oxidants and antioxidants indicate that improved growth and fertility of the fungus is likely caused by augmented reactive oxygen species levels triggered by the presence of phenolics.

## 1. Introduction

Lignocellulose breakdown by fungi is a complex and incompletely known process requiring a combination of enzymatic [1,2] and non-enzymatic reactions [3], whose ratios depend on the fungal species. Owing to the structure of the plant cell wall, removal or at least modification of lignin is often required to access the high-energy-containing cellulose [4,5,6,7]. Degradation of lignin is supposed to be energy-demanding and is construed as mostly non-stereospecific, although some bacterial enzymes have some stereospecific actions [8,9]. It requires the action of “auxiliary activity (AA) enzymes” [2]. These enzymes have various redox activities, including many generating peroxide (e.g., cellobiose dehydrogenases, glucose, other sugars and alcohol oxidases, etc.) and others using the peroxide to produce small reactive molecules (e.g., lignin peroxidases and Mn peroxidases). These small molecules then interact non-specifically with the various macromolecules present in the plant cell wall and cleave them. Nevertheless, additional enzymatic activities may be involved in lignin breakdown, such as laccases and β-etherases [1]. The non-enzymatic process relies on the hydroxyl radicals produced from the Fenton reaction to achieve the same end result. Fungi using the Fenton reaction fuel it by enzymatically producing peroxide [10].

It is most likely that having efficient enzymatic machinery towards lignin is not sufficient for effectively breaking down lignocellulose. Indeed, fungi must also breach the plant cell wall, which some species do by producing appressorium-like structures [11,12,13]. They must also resist the toxic compounds present in wood or generated during the degradation process [4,14]. Determining exactly how fungi break down lignocellulose will thus require a combination of biochemical, genetical and cytological analyses, which will thus be achieved only with well and easily handled models. Most studies on lignin breakdown are carried out with species belonging to the phylum *Basidiomycota* because these appear to be the most efficient ones to break down wood in nature. Unfortunately, most species of *Basidiomycota* are often difficult to manage in the lab, especially when gene deletions are envisioned. On the contrary, species belonging to the phylum *Ascomycota* are more easily handled and gene deletions are easier. Importantly, although not as efficient as in *Basidiomycota*, lignin degradation has been shown in some species inhabiting soil and wood, including *Xylaria* spp. [15], *Penicillium chrysogenum* [16,17], *Aspergillus flavus* and *Emericella nidulans* (=*Aspergillus nidulans*) [18], *Fusarium proliferatum* [19,20] and *Fusarium solani* [21]. Some *Ascomycota*, such as *Phoma herbarum*, even appear to be able to use lignin as a food source [22,23,24]. However, none of these ascomycetes has been used in gene deletion experiments to address lignin breakdown mechanisms.

Among the *Ascomycota*, a good model widely used to decipher how fungi degrade plant biomass is the coprophilous fungus *Podospora anserina* [12,25]. Targeted gene deletion in this species is easy [12,26] and its genome is replete with genes encoding AA enzymes [12,25]. Moreover, *P. anserina* is able to complete its lifecycle in the laboratory with many lignocellulose sources, including its natural growth substrate, herbivore dung and wood shavings as the sole carbon source [25,27,28]. This indicates that it can extract enough nutrients from the complex lignocellulosic sources to build the multicellular fruiting bodies involved in ascospore production, or perithecia, that it uses for dispersal. A recent study demonstrated that it is able to mineralize lignin [29]. In this study, the starting lignocellulose source, i.e., pre-isolated lignin from wheat straw re-associated with the hemicellulose fraction of the straw, while enabling fine analyses of the lignin hydrolysis, was not a natural food source for the fungus.

Here, we address the relationships between *P. anserina* and the lignin component of lignocellulose. First, we analyzed how this fungus interacts with various natural plant biomasses and show that it is able to utilize a wide range of lignocellulosic materials. We then confirm that it is able to break down and/or alter lignin by a combination of methods, including (UV/Vis) light spectroscopy, fluorescence analysis and pyrolysis–gas chromatography mass spectrometry (Py–GCMS). Finally, we report for the first time that *P. anserina* grows more and is more fertile in the presence of purified lignin and other polyphenolic compounds such as tannic acid, a phenomenon associated with the oxidative stress generated during lignin degradation, indicating that the fungus likely uses lignin and phenolics to signal its development.

## 2. Materials and Methods

### 2.1. Strains, Media and Chemicals

The *P. anserina* strains used in this study are the “S” (uppercase S) wild-type strain [30] used for sequencing [25,31] and the previously described *CAT^ΔΔΔΔΔ^* strain lacking its five genes encoding catalases [27]. The *Chaetomium globosum* strain was DSMZ 62,110.

Standard culture conditions, media and genetic methods for *P. anserina* have been described [26,32,33]. M2 medium is KH_2_PO_4_ 0.25 g/L, K_2_HPO_4_ 0.3 g/L, MgSO_4_/7H_2_O 0.25 g/L, urea 0.5 g/L, thiamine 0.05 mg/L, biotine 0.25 µg/L, citric acid 2.5 mg/L, ZnSO_4_ 2.5 mg/L, CuSO_4_ 0.5 mg/L, MnSO_4_ 125 µg/L, boric acid 25 µg/L, sodium molybdate 25 µg/L, iron alum 25 µg/L, dextrin 5 g/L, agar 12.5 g/L. M0 has the same composition except that dextrin is omitted. M4 has 5 g/L of crystalline cellulose CC4I (Whatmann) instead of dextrin. C. *Miscanthus giganteus* (MG) was provided by the B.E.S., Biomass Environment Systems group, a French association of farmers. Dried hay and hardwood shavings of undefined species were purchased from a pet store; oak and poplar sawdust were obtained from a sawmill and *Guibourtia demeusei* wood shavings from a brush factory. Fresh dung pellets from donkeys, dromedaries and rabbits were collected from the wild and stored at 4 °C before sterilization. Sterilization was achieved by autoclaving at 121 °C for 20 min.

Soluble lignin was from Sigma Aldrich (Cat. N° 471003: alkali lignin with low sulfonate content) as were insoluble lignin (Cat. N° 370959: alkali (kraft) lignin), tannic acid (Cat. N° 403040), humic acid (Cat. N° 53680), vanillyl alcohol (Cat. N° W373702) and syringyl (=2-6-dimethoxyphenol, Cat. N° D135550). Lignin purified from poplar, plane tree and wheat straw was kindly provided by Louis Monsigny [34] and oak wood extractive by Melanie Morel.

### 2.2. Growth and Fertility Assays

Growth and fertility was assayed on *mat+/mat-* heterokaryotic mycelia generated by grinding in 500 µL of sterile distilled water two plugs of 1 mm^3^, one from a fresh *mat+* mycelium and the other from a fresh *mat-* one, with a Fast-Prep apparatus (MP Biomedicals) as described [26,32,33]. Inoculation of 10 µL of the mix was conducted at the center of the various plates and vessels.

**Figure 1:**
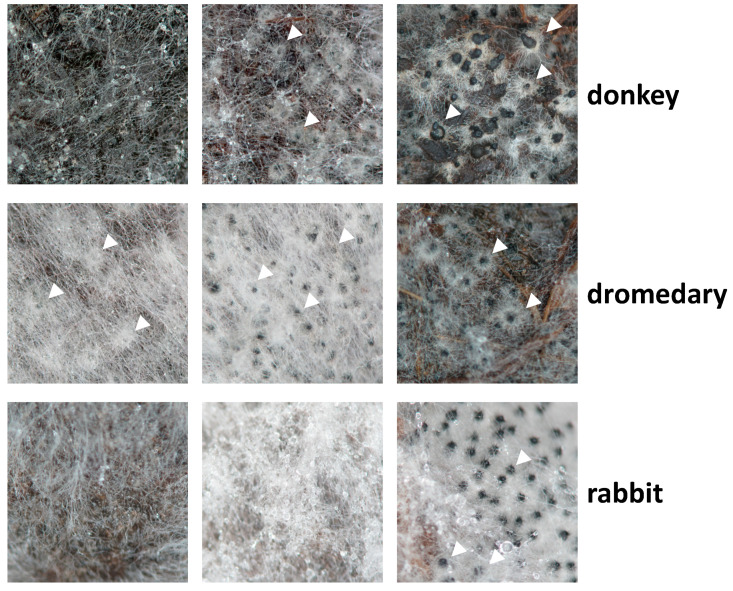
Growth and fructification of *P. anserina* strain on dung pellets of various origins. Pellets were inoculated with similar amounts of *mat+/mat-* heterokaryotic mycelia of strain S and incubated for one week, at which time the pictures were taken. Arrowheads point towards some of the fruiting bodies. Further incubation did not result in late appearance of fruiting bodies.

**Figure 2:**
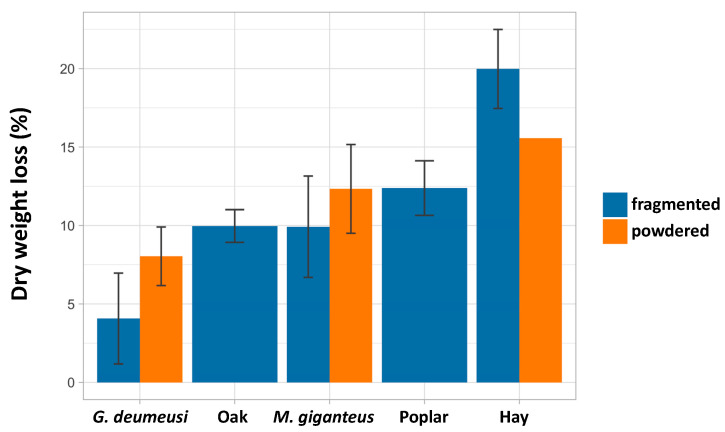
Dry weight loss in percent when *P. anserina* is grown on the indicated biomasses. The experiments are the combination of at least three assays, except for the powdered hay, which was assayed once.

**Figure 3:**
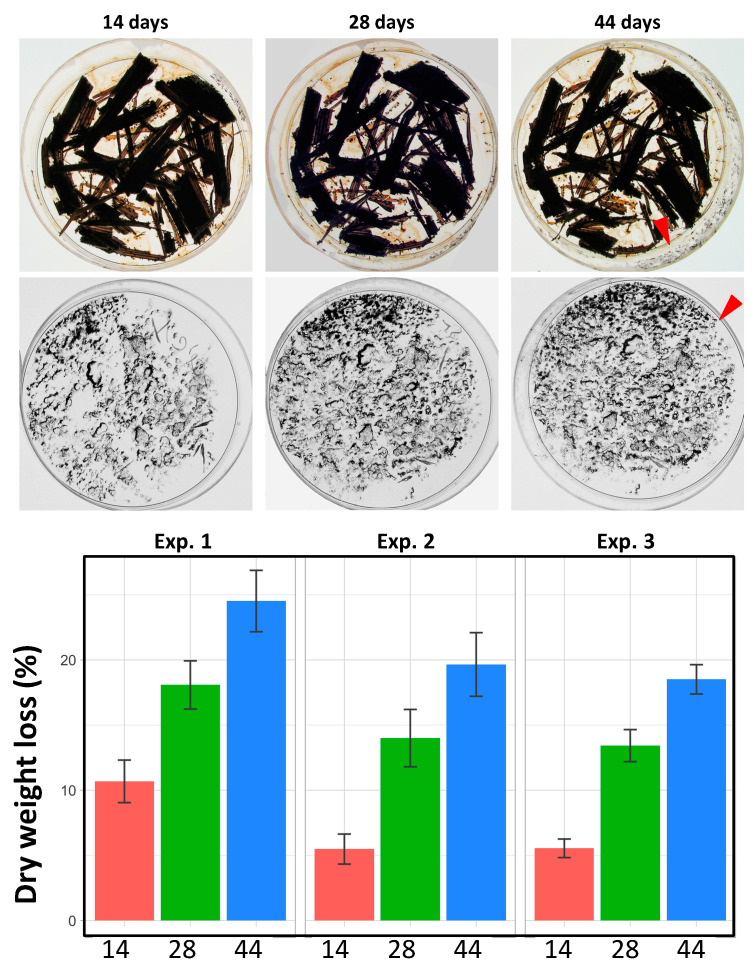
Dry weight loss of miscanthus after serial incubations. Top, scheme of the experiment. Middle, typical Petri plates after the first (14 days), second (28 days) and third (44 days) incubation. Perithecia are not easily visible owing to the darkening of the biomass triggered by drying. However, increased production of ascospores is easily visualized on the cover of the Petri plates. Red arrowheads point towards additional ascospores produced during the third incubation. Bottom, results of three independent experiments (in red, green and blue, each with 5 incubation plates) yielded the same outcome, with final loss ranging from 18% (Exp. 3) to 24.5% (Exp. 1).

**Figure 4:**
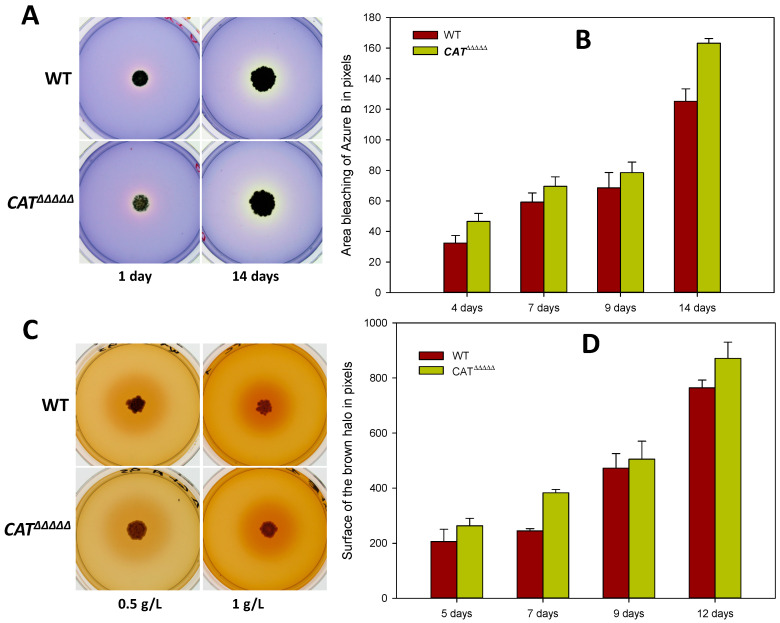
Discoloration of Azure B and transformation of lignin as a function of the incubation time. (**A**,**C**) Actual plates; quantification for Azure B bleaching (**B**) and lignin transformation (**D**) by *P. anserina* wild-type (WT) (brown bar) and *CAT^ΔΔΔΔΔ^* (green bar).

**Figure 5:**
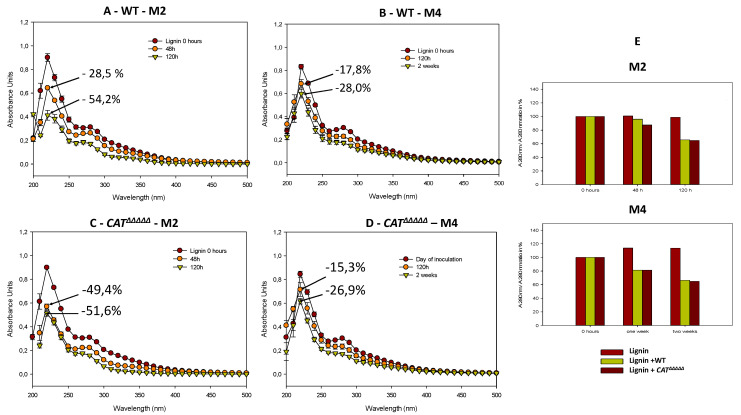
UV-visible absorption spectra of lignin (20 µM) in M2 medium in the presence of *P. anserina* wild-type (WT) (**A**) and *CAT^ΔΔΔΔΔ^* (**C**) at 0 h (brown circles) 48 h (orange circles) green triangle pointing down (120 h). UV-visible absorption spectra of lignin (20 µM) in M4 medium in presence of *P. anserina* wild-type (WT) (**B**) and *CAT^ΔΔΔΔΔ^* (**D**) at 0 h (brown circles) 120 h (orange circles) green triangle pointing down (2 weeks). (**E**) Absorbance at 280 nm at time “t” divided by absorbance at 280 nm at t zero hours (A280-t/A280-t0).

**Figure 6:**
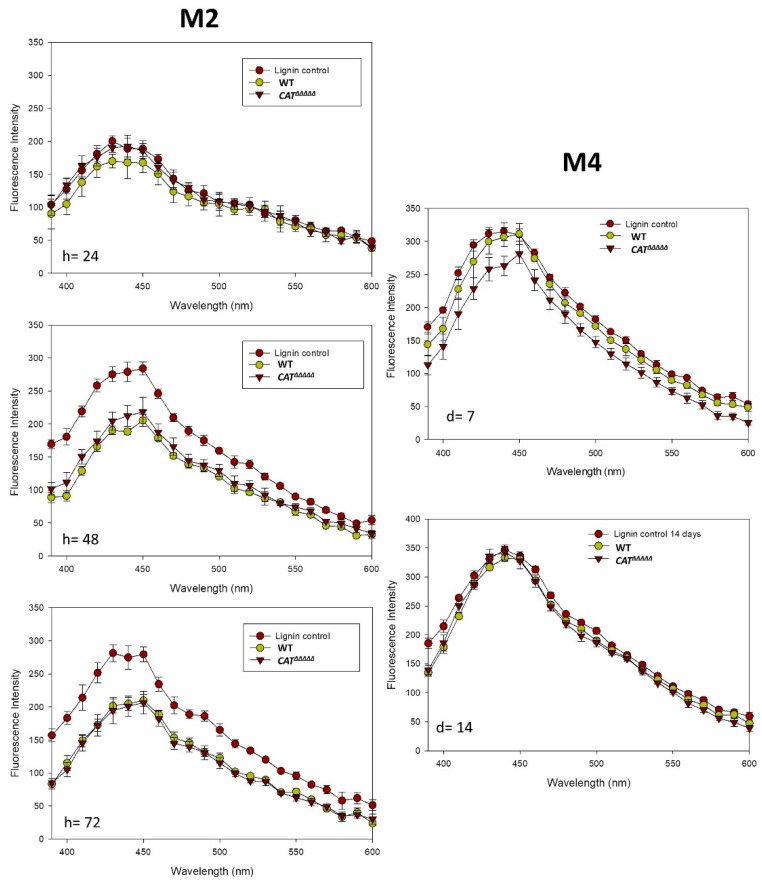
Fluorescence spectra with excitation at 355 nm recorded between 390 and 600 nm for lignin exposed to the fungus in M2 and M4 medium. Data are presented for lignin at 0 h in absence (brown circles) or in presence (green circles) of *P. anserina* wild-type (WT) after 24, 48 or 72 h compared to those obtained in presence of *CAT^ΔΔΔΔΔ^* (red triangle pointing down) after 24, 48 or 72 h of incubation in M2 medium or after 7 or 14 days of incubation in M4 medium.

**Figure 7:**
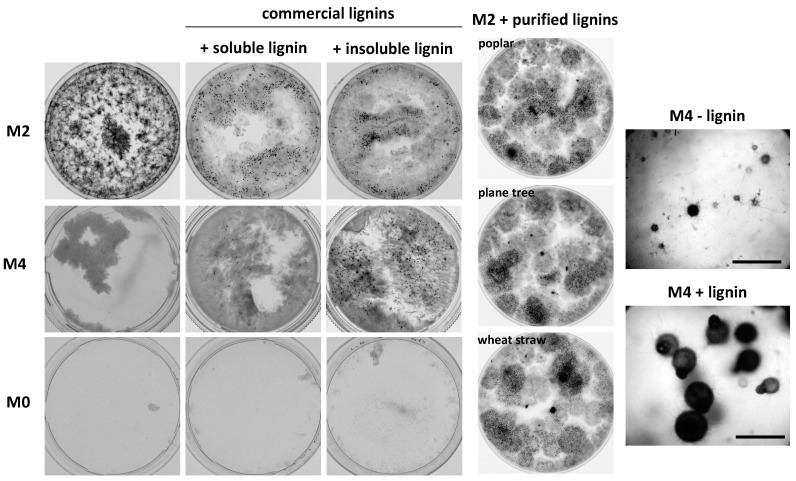
Fertility of *P. anserina* in liquid cultures in the absence or presence of lignin from various origins. Ø = 5-cm Petri plates were inoculated with 5 mL of M0, M2 or M4 medium supplemented or not with 0.04–0.1 g/L lignin (see text for optimal concentrations) with *mat+/mat-* heterokaryotic mycelium of the S strain and incubated for 10 days, at which time the pictures were taken.

**Figure 8:**
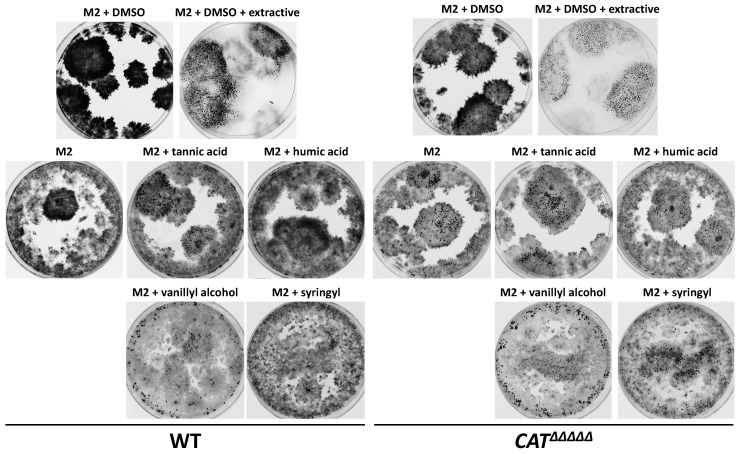
Fertility of *P. anserina* in liquid cultures in the absence or presence of phenolics. Ø = 5-cm Petri plates were inoculated with 5 mL of M2 medium supplemented with the indicated phenolics (see text for optimal concentrations) with *mat+/mat-* heterokaryotic mycelium of the S strain or *CAT^ΔΔΔΔΔ^* and incubated for 10 days, at which time the pictures were taken.

The dung pellets described in Figure 1 were incubated in humid chambers made with glass Petri plates. For the solid cultures of Figures S2 and S4, plates with Ø = 9 cm containing 27 mL of M2, M4 or 15 mL of cultures supplemented with the various solid biomasses were used. Note that in the plates with solid biomasses, the amount of jellified medium was lowered to avoid the complete submersion of the solid materials. For liquid cultures, plates with Ø = 5 cm contained 10 mL for growth and fertility assays (Figure 2, Figure 3, Figure 7, Figure 8, Figure 10 and Figure S4) and 5 mL for spectroscopy analyses (Figure 4, Figure 5 and Figure 6). Addition of sterile lignins and phenolics at concentrations ranging from 0.001 g/L to 1 g/L was carried out just before adding the fungus.

After a week of incubation at 27 °C in constant light, which is the minimal time required for perithecium maturation in optimal conditions (i.e., on M2 medium), the presence of fruiting bodies was monitored daily and their maturation ascertained by the presence of ascospores on the lids of the Petri dishes. Plates were incubated for up to two months to check if additional fruiting bodies appeared after prolonged incubation.

Dry weight loss was measured on at least three independent samples after drying the lignocellulosic materials remaining after 10 days of incubation overnight at 65 °C, except for the powdered hay sample of the experiment described in Figure 2, for which only one sample could be measured. Dry weight of mycelium was measured by collecting mycelia from five independent 40 mL volumes of cultures made with liquid M2 and five independent cultures made with liquid M2 supplemented with 0.04 g/L of soluble lignin incubated in 200 mL Erlenmeyers without agitation at 27 °C with constant light. Collection was made by filtration on pre-weighted paper pads and drying the pads overnight at 37 °C.

### 2.3. Azure B Discoloration and Lignin Transformation Time Course Assays

Petri dishes (Ø = 50 mm) were prepared with the following medium: sorbose 3 g/L; yeast extract 2 g/L and agar 10 g/L. The Azure B dye was added to the cooling medium at 25 mg/L just before pouring the plates. All the inoculations were carried out in triplicate and the Petri dishes were incubated at a temperature of 27 °C in the dark. After a few days, the fungi grew as a compact colony around which a halo of discoloration appeared. To quantify the capability of the investigated strains to bleach Azure B, photographs of the Petri dishes were taken at regular intervals. The images were then analyzed with Adobe Photoshop 6 software, which allowed quantification of the pixels corresponding to the uniform blue coloration of the starting medium versus those corresponding to the discoloration halo. The discolored surface in pixels (±standard deviation) was then plotted as a function of the incubation time.

Lignin transformation color assays were performed as Azure B discoloration, except that 0.5 or 1 g/L of lignin was added instead of the dye. Quantification followed the same methods by evaluating the dark brown versus light brown pixels.

### 2.4. UV-Vis Time Course and Fluorescence Time Course Assays

For UV-Vis time course assays, the fungus was inoculated in 7 mL of liquid M2 or M4 medium placed in 50 mm Ø Petri dishes. To this end, two plugs of the same size, one from a fresh *mat+* mycelium and the other from a fresh *mat-* one, were cut with a punch and ground in 500 µL of sterile distilled water with a Fast-Prep apparatus. The Petri dishes were inoculated with 10 µL of the mix. Lignin was added at the final concentration of 0.2 g/L. All the inoculations were carried out in triplicate and the Petri dishes were incubated at 27 °C in presence or in absence of light. Degradation of lignin was monitored daily with spectroscopy by following the absorbance and fluorescence emission of the lignin on an aliquot of 100 µL taken from each of the Petri dishes and diluted to 1/10 in the medium used for incubation. UV-Vis absorption spectra were acquired using a Perkin-Elmer Lambda^TM^ 25 UV-Vis spectrometer (Perkin Elmer, Akron, OH, USA). Spectra for each sample were collected between 200 and 500 nm spectral range at 1 nm spectral resolution with reference to M2 medium. The first point corresponded to the zero time and gave the value of the absorbance of the lignin at the beginning of the experiment. The UV-Vis absorption spectra were plotted in the absorbance (Absorbance Units) wavelength (nm) coordinates. A zero of the spectrophotometer was made with the medium without the fungus and the baseline was recorded. All the samples were then analyzed in the same order and in a room without direct light with medium M2 and M4 as reference. In the case of the medium M4 containing insoluble crystalline cellulose, the samples were centrifuged at 3500 rpm for 10 min to precipitate the cellulose and the absorbance measurements were carried out on the supernatants.

For the fluorescence time course assays, Petri plates with and without the fungus were generated as for the UV-Vis time course assays. Measurements of fluorescence were carried out in 96 well Greiner (Bio One) Surface black microplates, in a Tecan Infinite^®^ 200 microplate reader (Tecan Group Ltd., Männedorf, Switzerland) on 200 µL aliquots taken from every Petri dish and diluted to 1/10 in sterile distilled water prior to transfer in the black microplate. The fluorescence emission spectrum was recorded between 390 and 600 nm (with excitation at 355 nm). The entire assay was repeated every day. Fluorescence data were analyzed using the Excel software (Microsoft).

### 2.5. Determination of Ε-Molar Extinction Coefficient of Lignin

Successive dilutions of the lignin stock solution at 50 g/L were carried out in sterile Milli-Q^®^ (Merck Chimie SAS, an affliate of Merck KGaA, Darmstadt, Germany) water with concentrations ranging from 0.005 to 0.040 g/L. Absorbance of these dilutions was measured at the maximum absorption wavelength of the soluble lignin, i.e., at 220, 225, 237, 270, 280 and 287 nm. The absorbance values were plotted in the absorbance (absorbance units) lignin concentration (g/L) coordinates (Figure S1). The slope allows estimation of the value of the E-molar extinction coefficient of lignin. This parameter was then used to express the value of the degradation of lignin in moles per liter and per unit of time.

### 2.6. In Situ Pyrolysis–GCMS Analysis Method

Plates containing MG shredded in centimeter-sized pieces and MG grinded and sieved between 1 and 0.4 mm were added to M0 medium. The plates were then inoculated with *P. anserina* wild-type and incubated for two weeks until fertile fruiting bodies matured and expelled ascospores. Non-inoculated plates were treated in parallel as a control. Because fungal hyphae invaded the woody substrate, they could not be removed before analysis. A total of ten samples were studied (untreated control fragmented and grinded MG, four replicas of both fragmented and grinded MG treated with wild-type *P. anserina*). All samples weighed 0.2 mg. For each condition, three samples were analyzed. The data in Table 1 and Table 2 are expressed with plus and minus in a simplified qualitative way in order to rapidly compare the control versus the treated MG. Precise data are presented in Tables S1 and S2 as relative percentages of the different families of molecules detected by GCMS with standard deviations. Peak integrations were performed in the total ion chromatogram (TIC) using the Shimadzu software MDGCMS Solution. Percentages of peak area were obtained by dividing these peak integrations by the total pyrogram area for organic molecules. Areas were then grouped by families of molecules for each sample.

During the pyrolysis process, the different types of linkages are sensitive to different temperatures. The conversion of propyl chain begins at 180 °C [35,36], α-O-4 linkage can react at 200 °C [37], then β-O-4 can react at 245 °C [38,39]. Beyond 300 °C, C-C bonds in the alkyl chains are severed. Therefore, at lower temperatures, lignin is at first destabilized by cleavage of α-O-4 and β-O-4 bonds. After 300 °C, breakdown of lignin releases phenolic monomers as well as fractionation products such as methane, acetaldehyde or acetic acid [39,40]. Smaller alkyl side chains in the phenolic polymers are also cleaved, enabling the recovery of phenolic monomers possessing smaller side chains or devoid of them. Then, the fragmentation of methoxy groups in ortho position of hydroxyl groups occurs. Methane is then produced. When the temperature reaches 450 °C, only the phenyl linkage 5-5 and the ether linkage 4-O-5 are still present [41,42]. These linkages break between 500 and 800 °C. In this work, pyrolysis was operated under helium in an oven preheated at 400 °C, where small cupules containing the biomass were dropped. The biomass was instantaneously pyrolyzed and the evolved gases injected in the gas chromatography analyzer. This flash pyrolysis was important to assess what kind of reaction occurred during decomposition. Indeed, in this case, secondary reactions and charring were limited by the short residence time of the pyrolysate in the oven. This permitted an easier interpretation of the results compared to large-scale pyrolysis.

The pyrolysis of the different samples was performed with a pyrolyzer PY3030 Frontier Lab equipped with an Auto-Shot Sampler (type AS 1020 E) directly connected to a GCMS-QP2010Ultra SHIMADZU. The GC was equipped with a semi-polar capillary column ZB-1701 (30 m × 0.25 µm × 0.25 µm) with a (14%-cyanopropyl-phenyl-86%-dimethylpolysiloxane) phase. The EGA feature of the pyrolyzer with an Ultra Alloy^®^ capillary column (Deactivated tube L = 2.5 m, i.d. = 0.15 mm) was used to determine the optimal pyrolysis temperature for the MG samples. Evolved gas analysis (EGA) allows for the analysis of continuously volatile and pyrolyzate gases from the sample as it is heated. At the maximum of the plot of sample temperature vs. detector response (EGA thermogram), the optimal temperature can be read. Indeed, the gas emissions are at the highest point. In this sense, EGA is similar to thermal gravimetric analysis (TGA) but is performed with the same apparatus as the Py–GCMS analysis. Next, 0.6 mg of grinded MG was injected twice. The pyrolyzer heating method program began at 373.15 K for 10 min, followed by a heating rate of 15 °C/min for 40 min up to 973.15 K (for 5 min). The GC oven temperature was isothermal at 573.15 K. The optimal temperature was found to be 673.15 K (Figure S1).

Thermal pyrolysis–GCMS studies were used to determine chemical compound distribution in order to provide information on fundamental mechanisms of biomass degradation action due to fungal action. Three pyrograms were produced for each sample. Samples loaded in the autosampler were automatically dropped into the center of the pyrolysis micro-reactor, whose temperature was uniformly distributed. In order to limit secondary reactions, pyrolysis vapors were immediately injected into the GC. The pyrolysis was carried out at 673.15 K. A dynamic helium flow of 1.24 mL/min was continuously injected during the entire heating process. In order to provide selective compound separation, the GCMS heating method program began at room temperature (for 2 min) followed by a heating rate of 10 °C/min for 28 min up to 553.15 K (for 1 min). The temperatures of the injector and detector were set to 553.15 and 473.15 K, respectively. The ionization mode on the MS was electron impact. The mass range from m/z equal to 25 up to 600 was scanned and the identification of the compounds relied on the mass spectra library from NIST.

## 3. Results

### 3.1. Growth and Fertility of P. anserina on Dung Pellets Is Very Heterogeneous

Although *P. anserina* has sometimes been recovered from soil or as a plant endophyte, its main natural habitat is herbivore dung, from which it is frequently isolated. In nature, a succession of fungal species fructifies on dung, and *P. anserina* is among the fungi that fructify late in the succession, suggesting that it is able to scavenge energy from hard-to-digest materials such as the plant cell wall, which cannot be consumed by earlier fructifying species. As seen in Figure 1, when grown on sterilized dung pellets of various origins, the S strain of the fungus presents variable mycelium growth and fertility, suggesting that it encounters large variations in its growth substrate. These variations are likely related to the plant consumed by the herbivores, as they may contain different types of lignocelluloses and/or toxic chemicals of natural or anthropogenic origin. It is also possibly related to the type of herbivore, whose digestive tracts may break down plant materials differently and for different times. Additionally, dung is subjected to weather hazards, especially to rains that may wash low molecular weight compounds, providing further heterogeneity to *P. anserina* natural growth substrates.

### 3.2. P. anserina Is Able to Grow and Fructify on Various Lignocellulose Sources

To test the ability of wild-type *P. anserina* strain S to scavenge nutrients from diverse lignocelluloses, we assessed the production of fruiting bodies obtained up to two weeks after inoculation on various carbon sources (Figure S2, WT panel). On M2 medium with dextrin as sole carbon source, which is the minimal medium used routinely for cultivation, *P. anserina* produced a profuse mycelium that differentiated in one week a ring of mature fruiting bodies 1 cm away from the inoculation point and with a width of 1 cm. Dextrin was then replaced by several carbon sources composed of pure cellulose or various lignocelluloses, i.e., the M0 medium having the same composition as M2 but without dextrin was supplied with various insoluble celluloses or lignocelluloses. On M0 medium without any addition, *P. anserina* achieved only a spindly growth, barely visible to the naked eye, and was sterile. With 3 × 3 cm Whatman^TM^ (Cytiva Europe GmbH, succursale France, Velizy-Villacoublay, France) paper pads, fruiting body formation was also abundant and occurred with the same timing as on M2, but fruiting body formation was restricted to the paper pads and their neighboring areas. With cellophane, another form of amorphous cellulose, the *mat+/mat-* heterokaryons were unstable, rapidly creating numerous sterile sectors. However, fertility was high in the *mat+/mat-* heterokaryotic areas located at the center of the Petri dishes, with fruiting bodies differentiated as a more diffuse ring than on M2, but with the same kinetics. On the contrary, with crystalline cellulose as sole carbon source (M4 medium), fruiting body maturation was delayed by 3 days and the ring was also larger and more diffuse.

On more complex sources, fruiting body formation was delayed up to 10–12 days. Hay and MG were the preferred carbon sources since fruiting bodies matured the earliest (10 days) and were produced in large amounts both on whole hay/MG and on grinded hay/MG. Fertility was also high on *G. demeusei* wood provided either as wood shavings or as powdered sawdust. On the contrary, fertility was lower on oak and poplar sawdust. Especially, on poplar sawdust, few ascospores were expelled from the fruiting bodies. Finally, on two batches of wood shavings, fertility was poor and delayed, yielding also smaller fruiting bodies (batch n°1 obtained from a sawmill plant) or abolished (batch n°2 purchased in a pet store). Overall, this indicated that *P. anserina* is indeed able to scavenge nutrients to produce fruiting bodies from very different lignocellulose sources, although not with high efficiency from all of them.

### 3.3. Ability of P. anserina to Scavenge Nutrients from Lignocellulose Is Limited by a Developmental Program and Not by Inability to Further Break Down Lignocellulose

To evaluate how much nutrients *P. anserina* is able to scavenge, we measured the loss of dry weight after 15 days of fungal growth of strain S on some lignocelluloses. As seen in Figure 2, *P. anserina* is able to scavenge nutrients with varying efficiency from the different sources. It appeared most efficient with hay. However, hay, being dried grasses, still contains easy to digest materials such as cell contents, which overestimates the amount of nutrients scavenged from the lignocellulosic part. With the true woody lignocellulose, growth of *P. anserina* results in 5–15% dry weight loss. For *G. demeusei* and MG, fragmentation of the biomass resulted in a slightly higher weight loss, as expected if the fungus is able to access more easily the biomass. Note that there is not a direct correlation between the amount of consumed nutrients and the ability of the fungus to produce fruiting bodies. For example, *P. anserina* extracts as many nutrients from oak as from MG (Figure 2), yet it is much more fertile on MG than on oak (Figure S2).

Compared to brown and white rots that may degrade up to 70% of the biomass in a few weeks [43], the biomass consumed by *P. anserina* may seem modest. However, this species lives on an ephemeral substrate and may be adapted to produce fruiting bodies without having consumed all the nutrients that it may be able to retrieve, and then the fungus stops further development. Accordingly, prolonged incubations of the cultures mentioned in the previous section did not result in further production of fruiting bodies. To test the possibility that additional breakdown is possible, we made serial incubations of the same lignocellulosic material, interspaced with drying. Drying is of frequent occurrence in nature and *P. anserina* is able to withstand it. It was thus not necessary to inoculate again the fungus after drying, as it would renew growth upon rehydration. If a developmental program blocks production of perithecia, the fungus should be able to retrieve more nutrients from the biomass and thus produce more fruiting bodies. On the contrary, if the fungus exhausts its capacity to retrieve nutrients, it does not produce additional fruiting bodies upon further serial incubations. MG was thus incubated for two weeks with strain S of *P. anserina*, at which time the fungus had finished production of ascospores and consumed 5–10% of the biomass as measured by dry weight loss (Figure 3). The plates were then dried overnight at 65 °C and refilled with liquid M0. This resulted in new growth of the fungus, the production of another batch of ascospore-producing fruiting bodies and the further loss of 7–9% of dry weight (Figure 3). A third serial incubation following drying resulted in further 5–6% of weight loss and the production of further ascospore-producing fruiting bodies (Figure 3). At the end of the three incubations, *P. anserina* had reduced the MG biomass to 18–24.5% of its original weight in 44 days, showing that only a developmental program limits its efficiency to retrieve nutrients from lignocellulose. This indicated that *P. anserina* was likely able to consume more than the readily available hemicelluloses and cellulose present in MG.

### 3.4. Spectroscopic Analyses Indicate That P. anserina Efficiently Breaks Down Lignin

*P. anserina* is very efficient at retrieving nutrients from natural lignocellulose, likely because it is able to break down lignin [29]. We confirm here using alternative methods that it is able to alter the lignin components of the biomass, using first a color plate assay. Indeed, Figure 4A shows that *P. anserina* is able to discolor the Azure B dye. This dye is a structural analog of lignin often used to determine the activity of lignin peroxidase in fungi [44,45]. The degradation of this dye is a good indicator of *P. anserina*’s ability to degrade lignin. The presence of a dark brown halo surrounding the thalli when the fungus was grown in the presence of lignin on sorbose medium further substantiated an alteration of lignin by *P. anserina* (Figure 4B). Quantification of the halo evidenced a gradual increase during the 7 days of incubation. This experiment also evidenced the inhibitory effect of lignin on growth since the diameter of the brown halo was 44.25% smaller for the 1 g/L concentration than for the 0.5 g/L one.

To further validate the degradation of lignin by *P. anserina*, we quantified the UV absorption spectrum of lignin in M2 or M4 medium before and after incubation of the fungus (Figure 5). These two media differ by the nature of the carbon source (dextrin for M2 and crystalline cellulose for M4) and allow different growths and fertility (Figure S2). Crystalline cellulose being more recalcitrant to digestion may also trigger the release of more enzymes involved in lignocellulose breakdown. The soluble lignin (Sigma Aldrich cat n° 471003) UV-absorption spectrum was simple in shape and it contained two weak asymmetrical bands: the first was in the region of 210–230 nm, the second with a maximum at 280 nm. This second band indicates the presence of benzene rings in the molecule [46]. On the day of inoculation by the wild-type strain, the lignin concentration, based on the calculation of the molar extinction coefficient (Figure S1), is equal to 20 µM. The concentration of lignin decreased by 28.52% in 48 h and 54.18% in 120 h (Figure 5A). The lignin concentration was thus drastically reduced by half in 5 days in M2. Fine analysis of the spectra (Figure 5A) shows that, when lignin is degraded, the peaks at 220 and 280 nm decreased in intensity and that the peak at 280 nm shifted at 270 nm, revealing thus a hypsochromic effect, probably due to the modification of the resonances of the benzene rings [47]. On M4, the same phenomenon occurred but was delayed by several days, since the absorbance of lignin at 220 nm decreases by 28% after only 2 weeks (Figure 5B). This further showed that lignin was altered by the fungus.

We also analyzed the fluorescence spectrum with UV excitation at 355 nm of lignin before and after adding the fungus (Figure 6). The shape of the emission spectra of lignin alone was quite simple, with a peak around 430 nm, as observed elsewhere [48]. At the time of inoculation, no real differences between the lignin spectra were observed with or without the fungus. However, in M2 medium after 48 and 72 h of incubation, the intensity of the fluorescence of a large peak comprised between 410 and 460 nm decreased by around 23–25% in the sample treated with the fungus when compared to the control without *P. anserina*, arguing again that the fungus modified the chemical structure of lignin. The same result was observed in the M4 medium with a delay of a few days. According to Barsberg et al. [48], the decrease is due to the interaction between free quinones, either produced by the fungus or released by lignin degradation, and lignin. We can thus tentatively propose that the modification of the UV spectrum showing a hypsochromic shift in the peak from 280 to 270 nm corresponded to the loss of benzene rings coupled with the decrease in fluorescence of the lignin interacting with quinones.

Calculation of the absorbance ratio at 280 nm, which monitors chemical [49] and biological [50] degradation of lignin, allowed confirmation that the fungus likely acted in part by breaking benzene rings within lignin. Indeed, the ratio of absorbance at 280 nm at time “t” divided by absorbance at 280 nm at t zero hours (A280-t/A280-t0) decreased slightly after 48 h in M2 and by around 40% after 120 h (Figure 5E). In M4, the decrease was around 20% after a week, and 30% after two weeks.

### 3.5. Pyrolysis–Gas Chromatography Analyses Confirm That P. anserina Modifies Lignin When Growing on MG

To confirm that *P. anserina* breaks down lignin and to obtain further insight into how the fungus alters lignin when growing on lignocellulosic materials, we compared by Py–GCMS MG untreated and treated for two weeks by the fungus. This method detects decomposition products of lignocellulose [51] and therefore gives an insight into the effect of fungal growth on its structure [52,53,54,55].

Pyrolysis of untreated MG and MG treated with wild-type *P. anserina* released the families of compounds listed in Table 1 and Table 2 for qualitative data and Tables S1 and S2 semi-quantitative ones, respectively. Grinded and fragmented MG analyses yielded the same qualitative results, with a higher standard deviation for the fragmented samples, as expected from the higher heterogeneity of the starting material. Comparison of the untreated MG with the one treated with the wild-type fungus showed that pyrolysis released, on average, more phenolic monomers when MG was treated, indicating that *P. anserina* was able to break down lignin [56,57]. The linear fractionation products like acetic acid were globally in lower amounts (Table 1 and Table 2, Tables S1 and S2). Furanics were statistically difficult to analyze but their sum with the other monocyclic oxygenated compounds clearly increased (Tables S1 and S2). The phenolic compounds with a side chain smaller than three carbons increased significantly, while the C3 phenolics were kept to similar amount, except for grinded MG S5 and fragmented S3, in which an increase was observed. The non-phenolic compounds derived from lignin were mainly molecules with methoxy groups but without hydroxyl or polycyclic aromatic hydrocarbon (PAH), harder to identify precisely by GCMS. The decrease in the treated samples suggested that the hydroxyl is more easily preserved and smaller molecules are formed. Anhydrous sugars that resulted from the pyrolysis of cellulose and hemicellulose seemed to slightly decrease in treated samples. Overall, this confirmed that *P. anserina* altered the structure of lignin by potentially cleaving the β-O-4 linkages of lignin [58] and other aryl ether linkages, as proposed by van Erven et al. ([29]; Figure S3). This scheme is compatible with the lower amounts of linear compounds like acetic acid from side chains, the decrease in non-phenolic compounds derived from lignin and the more efficient release of phenolic monomers. Weakened lignin was also compatible with the observation of a relatively decreased amount of anhydrous sugars in emitted gases and increase in cyclic oxygenated compounds probably due to the rupture of osidic bonds in cellulose and hemicellulose [56].

### 3.6. P. anserina Grows and Fructifies Better in the Presence of Lignin and Oak Extractives

During spectroscopy experiments in which *P. anserina* was grown in liquid cultures, we noticed by visual inspection that the fungus grew better and fructified much more in the presence of lignin (Figure 7). This effect was seen in two different media (M2 and M4) and with five different kinds of lignin, including highly purified insoluble lignin from poplar, plane tree and wheat straw. To evaluate the growth improvement, we measured the dry weight of the fungus in the presence and absence of 0.04 g/L soluble lignin in M2 medium. We observed two to three times more biomass after three days of growth (28.2 ± 2.8 mg per 40 mL cultures in the presence of lignin versus 10.6 ± 2.2 mg in its absence; n = 5 for each condition), confirming a huge improvement in growth. When assayed in jellified Petri plates, we did not observe such an effect, finding only a toxicity of lignin at high concentrations (>1 g/L). The minimum lignin concentration in M2 to observe good growth and fertility was 0.04 g/L for the soluble commercial lignin and 0.01 g/L for the insoluble one. These low values make it unlikely that improved growth and fertility were solely linked to contaminating nutrients present within lignin. This was confirmed by the lack of extensive mycelium growth when lignin was added to M0, although, as previously reported, the fungus grew slightly better in the presence of lignin ([25]; Figure 7).

Intriguingly, oak wood extractives at a concentration of 0.05 g/L had the same effect as lignin in promoting growth and fertility (Figure 8). Wood extractives contain in varying amounts both aliphatic/alicyclic and phenolic compounds [14]. Because lignin is also composed of phenolic compounds, we tested whether tannic acid, a frequent polyphenol of wood extractive, could improve growth and fertility. As seen in Figure 8, 0.01 g/L of tannic acid greatly improved growth and fertility. To test if the degradation products of lignin could also improve growth and fertility, we tested humic acid, vanillyl alcohol and syringyl (2-6-dimethoxyphenol) (Figure 8). Among these, vanillyl alcohol was very efficient in restoring growth and fertility at concentrations as low as 0.005 g/L, while humic acid restored only growth at concentrations higher than 0.02 g/L and syringyl restored growth and fertility at concentrations higher than 0.1 g/L. Therefore, many phenolics connected to the biosynthesis and degradation of lignin and tannins were able to improve the growth and fertility of *P. anserina* when cultivated in liquid cultures.

Because growth and fertility improvements by polyphenols were specifically seen in liquid media, in which the fungus grows within the liquid, and not in solid media, in which it grows mostly at the interface between liquid and air, we surmise that oxidative stresses could be involved in promoting fertility in the presence of lignin. We thus tested the effect of various oxidative stresses and antioxidants on growth and fertility improvement, starting with an analysis of the *CAT^ΔΔΔΔΔ^* mutant lacking the five genes encoding catalases in *P. anserina* [27].

### 3.7. The CAT^ΔΔΔΔΔ^ Catalase Mutant Is Able to Grow and Fructify on MG and in Liquid M2

**Figure 9:**
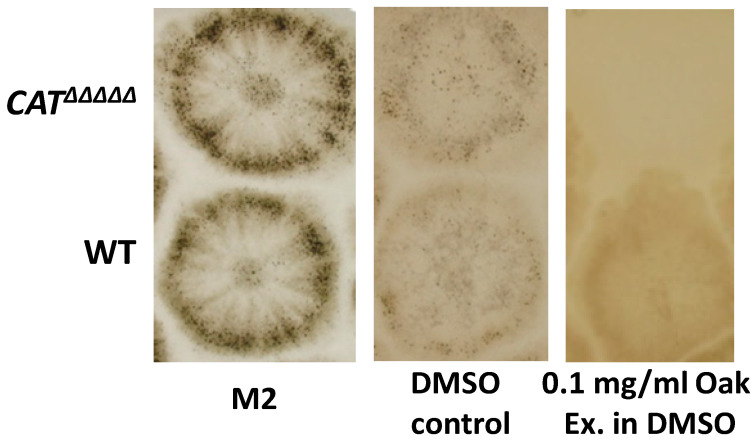
Sensitivity to wild-type and mutants to oak extractables. Extractables are soluble in dimethyl sulfoxide (DMSO) and the plate with the same amounts of DMSO but lacking extractables (DMSO control) are used as control.

Previous analyses have shown that catalases, especially the putatively non-secreted CAT2 enzyme, which breaks down hydrogen peroxide into water and oxygen, are crucial for growth on pure lignin and perithecium development on wood shavings ([27]; Figure S2 *CAT^ΔΔΔΔΔ^* panel). These enzymes are, on the contrary, dispensable on medium lacking lignin, including those having paper, dextrin or glucose as the sole carbon source ([27]; Figure S2 *CAT^ΔΔΔΔΔ^* panel), suggesting that they are required specifically in the presence of lignin. This is confirmed by the fact that *CAT^ΔΔΔΔΔ^* does not grow on medium containing high levels of lignin—a defect that is corrected by the addition of exogenous bovine catalase [27]. Intriguingly, *CAT^ΔΔΔΔΔ^* was able to grow and reproduce on MG to the same extend as the wild-type (Figure S2). Notably, the growth and fertility of *CAT^ΔΔΔΔΔ^* were lower than those of the wild-type on hay (Figure S2), which, like MG and unlike the other tested woody materials, is composed of mostly monocotyledon biomass. Monocots are known to have a lignin structure different from dicots [59,60]. It is thus not clear what differentiates MG from all the other biomass sources. Possibly, MG triggers the production of less hydrogen peroxide by the fungus, resulting in its survival, as *CAT^ΔΔΔΔΔ^* is hypersensitive to this molecule [27]. It may alternatively be the level of extractives, as, unlike the wild-type, the *CAT^ΔΔΔΔΔ^* mutant could not grow on medium containing 0.1 mg/mL of oak extractives (Figure 9). Interestingly, the growth of *P. anserina* on dried hay clearly triggered the release of a yellowish-pigmented compound that was not released in the *CAT^ΔΔΔΔΔ^* mutant cultures (Figure 2). Overall, these data show that catalases are required only on some lignocelluloses, including those containing high levels of extractables.

Evaluation of the ligninolytic activity of the *CAT^ΔΔΔΔΔ^* mutant showed only small differences when compared to the wild-type. Discoloration of the Azure B dye by *CAT^ΔΔΔΔΔ^* increased with incubation time (Figure 4A,B). This effect became significant after 2 weeks of incubation, at which time the discoloration halo of the wild-type was 30% less important than that of *CAT^ΔΔΔΔΔ^*. Quantification of the dark halo in the presence of lignin in the sorbose medium indicated a more intense coloration and a slightly larger halo, indicative of an effect of *CAT^ΔΔΔΔΔ^* 12% higher when compared to the wild-type (Figure 4 C,D). Under the action of *CAT^ΔΔΔΔΔ^*, the decrease in absorbance at 220 nm was 49.40% in 48 h and only 51.60% in 120 h on M2 (Figure 5C), indicating a faster degradation of lignin by the mutant. However, ratio of absorbance at 280 nm as a function of time evolved similarly (Figure 5E) and fluorescence spectrum analyses did not reveal statistically significant differences between the wild-type and the mutant in M2 after 48 or 72 h (Figure 6). Moreover, the values on M4 for the decrease in absorbance at 220 nm were nearly identical for both the wild-type and *CAT^ΔΔΔΔΔ^* (17.8% vs. 15.3% at 120 h and 28.0% vs. 26.9% at two weeks, Figure 5D).

Interestingly, *CAT^ΔΔΔΔΔ^* was able to grow better than the wild-type and to produce perithecia in liquid M2 (Figure 8). Moreover, all compounds identified as restoring growth and fertility of the wild-type did so for the *CAT^ΔΔΔΔΔ^* mutant, but with a stronger effect at the same concentrations (Figure 8). Overall, this argued for a role of oxidative stress generated by the presence of lignin and other polyphenols in restoring the growth and fertility of *P. anserina* in liquid cultures.

### 3.8. Oxidative Stress Promotes Growth and Fertility of Liquid Cultures in the Presence of Lignin

**Figure 10:**
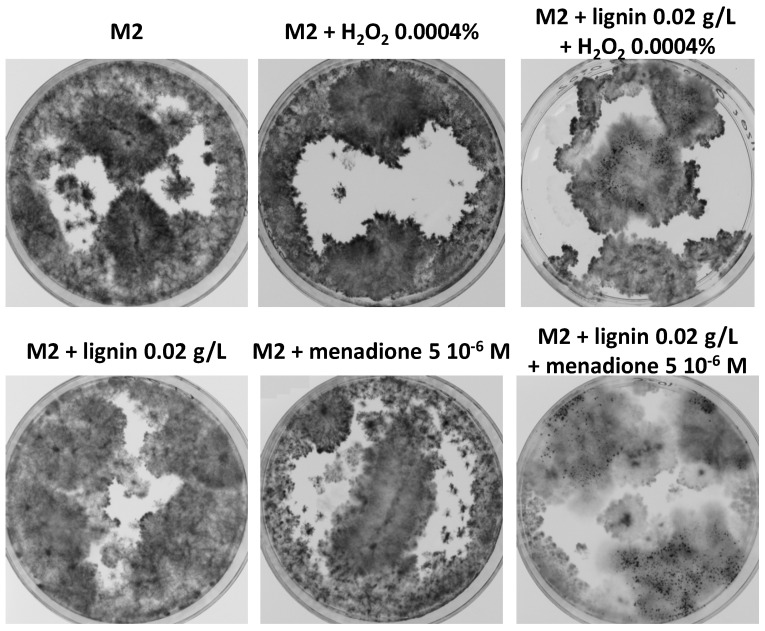
Synergistic effect of lignin and reactive oxygen species on fertility of *P. anserina* in liquid cultures. Ø = 5-cm Petri plates were inoculated with 5 mL of M2 medium supplemented with the indicated chemicals with *mat+/mat-* heterokaryotic mycelium of the S strain and incubated for 10 days, at which time the pictures were taken. Fruiting bodies (visible as small black dots) are only formed in the presence of lignin and menadione or lignin and H_2_O_2_.

To directly test if oxidative stress promoted the growth and fertility of liquid culture, hydrogen peroxide (H_2_O_2_) and menadione, a quinone generating superoxide by auto-oxidation in a redox-cycling reaction within the cells, were added to cultures of wild-type *P. anserina* in the presence or in the absence of lignin. In the absence of lignin, no improvement in growth and fertility was observed at low concentrations (0.0004% for H_2_O_2_ and 5 × 10^−6^ M for menadione), while at higher concentrations, both chemicals were toxic and inhibited growth (Figure 10). Interestingly, in the presence of 0.02 g/L of soluble lignin, which did not promote fertility but only a slight growth improvement, 0.0004% H_2_O_2_ and 5 × 10^−6^ M menadione triggered the development of fruiting bodies. Hence, oxidative stress is able to promote growth and fertility in the presence of lignin, confirming the notion that improved growth and fertility is connected to the production of reactive oxygen species.

### 3.9. Antioxidants Do Not Inhibit Improved Growth and Fertility by Lignin

Intriguingly and paradoxically, while oxidative stress seems to be involved in the growth and fertility improvement of liquid culture containing phenolics, most of them have been shown to harbor antioxidant activities in vitro [61,62,63,64,65,66]. We therefore tested whether the addition of antioxidants would improve or diminish the growth and fertility of liquid cultures. We tested the fertility of liquid cultures containing no lignin or 0.004 g/L and 0.1 g/L of soluble and insoluble lignin in the presence of L-ascorbic acid in concentrations ranging from 0.004 to 0.1 g/L and catalase from 1000 to 100,000 u/mL. We did not observe any modification of fertility compared with the control containing no antioxidant in all tested conditions.

### 3.10. Improved Fertility in the Presence of Lignin Is Also Observed in Chaetomium globosum

To evaluate whether growth and fertility improvement in the presence of lignin was restricted to *P. anserina* or could be present in related fungi, we assayed the growth and fertility of *Chaetomium globosum*, another *Sordariales* fungus, in the presence of lignin. As seen in Figure S4, soluble and insoluble lignin improved both growth and fertility in solid and liquid cultures, showing that its action on development is not restricted to *P. anserina.*

## 4. Discussion

At the present time, a complete picture of how any fungus degrades plant biomass is not available because of the complexity of the degradative process that calls for dozens of enzymes [1,67,68], the necessity for the fungus to resist lignin degradation products and other chemicals present in plant biomasses (e.g., extractives), which also likely involves numerous enzymatic systems [4,14] and the contribution of appressorium-like differentiation in the breaching of the plant cell walls [13]. Deciphering this process would be greatly facilitated by using a species that is able to degrade lignin and is easy to manipulate in the lab, especially with an efficient genetic analysis. Here, through a combination of techniques, we confirm that the ascomycete fungus *P. anserina* can be such a model. It can use numerous biomasses with varying degrees of efficiency to grow and fructify. We confirm that it is able to degrade lignin. It also differentiates appressorium-like structures involved in biomass penetration [11,13]. Overall, this fungus presents a large array of mechanisms to efficiently breach and degrade lignocellulose. This fungus is easy to handle in the lab, especially when forward and reverse genetic analyses are envisioned. As previously underlined [12,26], this fungus is thus a good candidate to decipher mechanisms of biomass degradation by fungi. Note that although basidiomycetes are deemed more efficient than ascomycetes when lignin degradation is concerned, several studies have shown that many ascomycetes can break down lignin and possibly even retrieve nutrients from it [15,16,17,18,19,20,21,22,23,24], although most cannot extract large amounts of food from lignocellulose. Here, we show that the limitation in the amounts of nutrients retrieved by *P. anserina* is likely not due to limitation in its lignocellulose degradation machinery, but rather to a developmental program. It remains to test whether other ascomycetes are limited in a similar fashion. Owing to the fact that these fungi are the most abundant in soils [69], in which successions of growth and rest may be frequent, it is thus possible that they have in nature a role much more important than usually described in the recycling of lignocellulose into carbon dioxide.

The data presented here confirm that the interaction of *P. anserina* with various lignocelluloses is complex. Even on its preferred natural growth substrate, dung, we detect large variations in the amounts of mycelium and fruiting bodies that the fungus produces. The reasons for this are likely multifactorial and include the secretion of different enzymatic cocktails, as previously demonstrated [29,70,71]. It was shown that the presence of lignin reduces the secretion of cellulases and galactomannanases and increases that of laccases and H_2_O_2_-producing enzymes, which should favor ligninolysis [29]. Crucial roles of laccases and catalases in the ability of the fungus to grow and fructify on wood shavings were previously demonstrated [27,72,73]. The presence of various substances may also modulate growth and fertility. Surprisingly, these do not only act though deleterious effects. Indeed, the addition of phenolic compounds including lignin, extractives and tannins, their precursors and degradation products promotes growth and fertility with varying levels of efficiency, likely in conditions of low oxygen availability (here mimicked by cultivation in liquid). How this regulation works is not clear, especially because deleterious effects may antagonize growth and fertility improvement. On the one hand, the *CAT^ΔΔΔΔΔ^* mutant shows higher growth fertility than the wild-type in liquid cultures in the presence of phenolics, suggesting that H_2_O_2_ signaling participates in growth and fertility improvement. On the other hand, *CAT^ΔΔΔΔΔ^* exhibits lower fertility on all biomasses, except on MG. This could be accounted for by the lower resistance of the mutants to H_2_O_2_, which may counteract the positive effects of phenolics. However, why lower resistance to H_2_O_2_ is not important in miscanthus is unclear. Possibly, H_2_O_2_-producing enzymes may not be secreted in large amounts on this biomass. Intriguingly, phenolics that improve the growth and fertility of the fungus are known antioxidants, yet they act through synergic effects with reactive oxygen species. This tentatively suggests that they may act through the activation of specific pathway(s), rather than having general roles in modulating the redox status of the substrate. This is substantiated by the fact that lignin modulates transcription in *P. anserina* [74]. Interestingly, improved growth and fertility triggered by lignin may be a general phenomenon in fungi, since it was previously demonstrated that lignin increases growth and fruiting body production in the basidiomycete shitake [75] and that extractives stimulate the production of perithecia in *Chaetomium indicum* and *Chaetomium globosum* [76], two fungi closely related to *P. anserina*. The culture assays in the presence of lignin presented here confirm that lignin is indeed able to improve the growth and fertility of *C. globosum* (Figure S4).

We develop here several methods, including some easy to implement for large-scale assays, to show that *P. anserina* degrades lignin. Indeed, simple tests in spectrophotometry (UV/Vis) and fluorescence as well as discoloration assays on Petri dishes, all relatively easy to set up, allowed us to highlight the degradation capacity of lignin by *P. anserina*.

Through Py–GCMS, we confirm the data of van Erven et al. [29] showing that the β-O-4 linkages are likely the preferred target for breakdown also on a natural substrate. Lignin is a complex polymer originating from the polymerization of three monolignols: coniferyl, sinapyl and p-coumaryl alcohols (Figure S3). In wood, lignin bonds are mainly of the β-O-4 aryl ether linkage type (30–40% in softwoods and 40–50% in hardwoods [77]). In the case of MG [78], they represent 82–84% of the bonds; the rest involve resinol (6–7%) and phenyl-coumaran bonds (10–11%). The main structures identified in MG lignin as defined by [79] are presented in Figure S3. The increased release of lignin monomers after the growth of *P. anserina* is compatible with the cleavage of the β-O-4 linkages by the fungus and potentially other ether aryl linkages (α-O-4, 4-O-5) (Figure S3). A fungus closely related to *P. anserina* has previously been identified as being able to perform the cleavage of these β-aryl ethers [58]. The involved enzyme machinery is still unknown, but it has been shown in bacteria that three enzymes, a Cα-dehydrogenase, a β-etherase and a glutathione lyase, are sufficient to cleave β-O-4 linkages [80,81]. However, *P. anserina* does not appear to possess all of these enzymes. While its genome encodes four proteins similar to bacterial Cα-dehydrogenases (Pa_1_3450, Pa_1_21060, Pa_3_7320 and Pa_4_7010), *P. anserina* apparently lacks enzymes resembling the bacterial β-etherases and glutathione S-transferases involved in the cleavage of the β-aryl ether. Its genome encodes, however, at least one protein similar to glutathione S-transferases of the GTE family postulated to be involved in lignin breakdown (Pa_1_1290 and possibly Pa_3_10450; [82]). Determination of whether these proteins are actually involved in lignin breakdown awaits more analysis.

## 5. Conclusions

Ascomycete fungi are usually thought to be of minor importance for lignin degradation. Here, we confirm that the model ascomycete *Podospora anserina* efficiently breaks down lignin but is limited in doing so by a developmental program. We also show that lignin and other polyphenolic compounds improve the growth and fertility of the fungus when grown in liquids, likely by promoting an oxidative stress. This argues that the fungus uses lignin and its degrading products, as well as other phenolics present in plant cell walls, to signal various stages of its development.

## Figures and Tables

**Table 1:** Effect of fungal treatment on targeted molecules and groups of molecules. Control vs. samples in powder treated with *P. anserina* S *mat+/mat-* strain (S1–S5).

Powdered MG	Control MG Raw	S1	S2	S3	S4	S5
*Group of compounds*						
furanics	16.7%	-	+	-	+	+
phenolics Cn < 3	17.5%	+	+	++	++	++
phenolics C3	5.3%	-	=	=	=	++
total phenolics	22.8%	+	+	++	++	++
other lignin derived compounds	5.6%	=	-	-	--	--
anhydrous sugars	6.4%	-	-	-	-	--
linear compounds	40.2%	-	--	-	-	-
cyclic compounds	8.4%	++	++	+	++	++
acetic acid	16.3	-	-	-	-	--

++: increase statistically confirmed --: decrease statistically confirmed; +: average tendency to increase -: average tendency to decrease; =: mean value difference < 0.4.

**Table 2:** Effect of fungal treatment on targeted molecules and groups of molecules. Control vs. samples in fragments treated with *P. anserina* S *mat+/mat-* strain (S1–S5).

Fragmented MG	Control MG Raw	S1	S2	S3	S4	S5
*Group of compounds*						
furanics	14.9%	=	+	--	--	=
phenolics Cn < 3	18.4%	++	++	+	++	++
phenolics C3	3.9%	-	=	++	-	=
total phenolics	22.3%	+	++	++	++	++
other lignin derived compounds	3.2%	++	=	+	++	--
anhydrous sugars	4.6%	-	-	=	--	-
linear compounds	44.7%	--	--	--	--	-
cyclic compounds	10.2%	++	++	++	++	++
acetic acid	15.7%	--	--	--	--	-

++: increase statistically confirmed --: decrease statistically confirmed; +: average tendency to increase -: average tendency to decrease; =: mean value difference < 0.4.

## Supplementary Materials

The following are available online at https://www.mdpi.com/2309-608X/6/4/278/s1, Figure S1: Calibration curves and optimal temperature determination for Py–GCMS. Figure S2. Fertility of *P. anserina* on various lignocelluloses. Figure S3. Lignin structure. Figure S4: Fertility of *Chaetomium globosum* in the absence and presence of lignin. Table S1: Semi-quantitative analyses of fractionation products released from grinded MG, Table S2: Semi-quantitative analyses of fractionation products released from fragmented MG.
