# Supplementary material for: Lignin Degradation and Its Use in Signaling Development by the Coprophilous Ascomycete Podospora anserina
Source: J Fungi (Basel). 2020 Nov 11;6(4):278. doi: 10.3390/jof6040278 (PMC7712204; doi:10.3390/jof6040278)
Supplement: Supplementary file 1 [file jof-06-00278-s001.zip › jof-945128-supplementary/Figure S1.pdf]

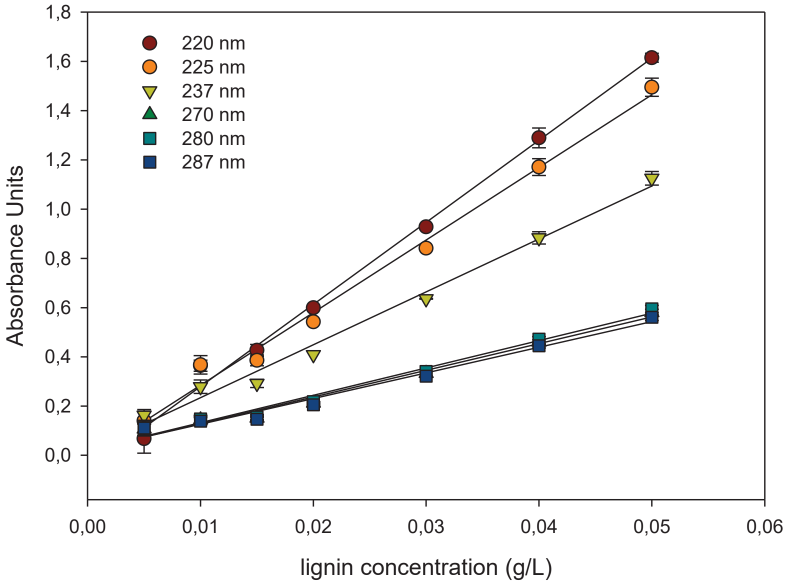

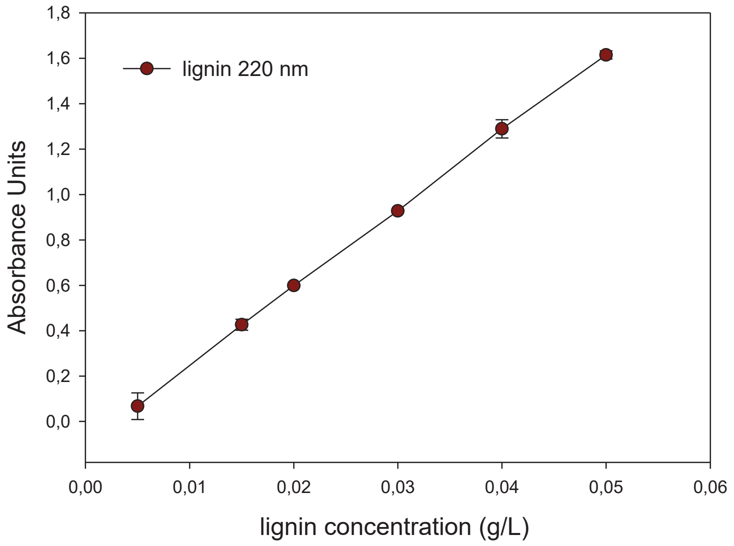

Figure S1: Calibration curves for the chosen wavelengths (225, 237, 270 and 280nm) are shown for the same concentration of lignin (0.02 g/L) (Sigma ref 471003, MW ~10 000 Da). Based on the experimental data of Lee et al., (2013), calibration graphs were plotted for bands at λ max = 220, 237, 270 and 280 nm (Fig. 6). The graphs indicate that the absorbance is a linear function of lignin concentration for all of the bands.

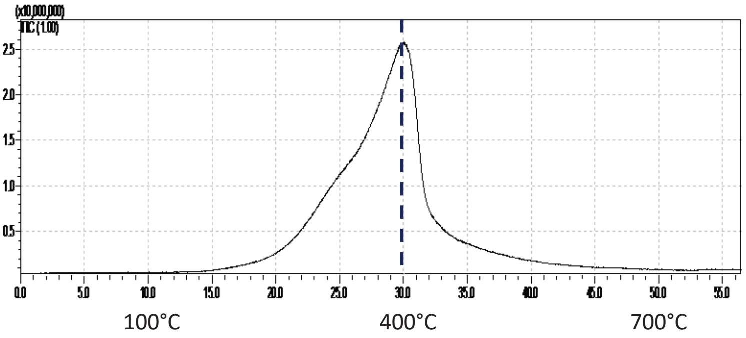

Time (min.)	10.00	30.00	50.00
Temperature (°C)	100.00	400.00	700
Temperature (K)	373.15	673.15	973.15

Figure S2: Optimal temperature determination for Py-GC/MS.
