# Supplementary material for: Lignin Degradation and Its Use in Signaling Development by the Coprophilous Ascomycete Podospora anserina
Source: J Fungi (Basel). 2020 Nov 11;6(4):278. doi: 10.3390/jof6040278 (PMC7712204; doi:10.3390/jof6040278)
Supplement: Supplementary file 1 [file jof-06-00278-s001.zip › jof-945128-supplementary/Figure S2.pdf]

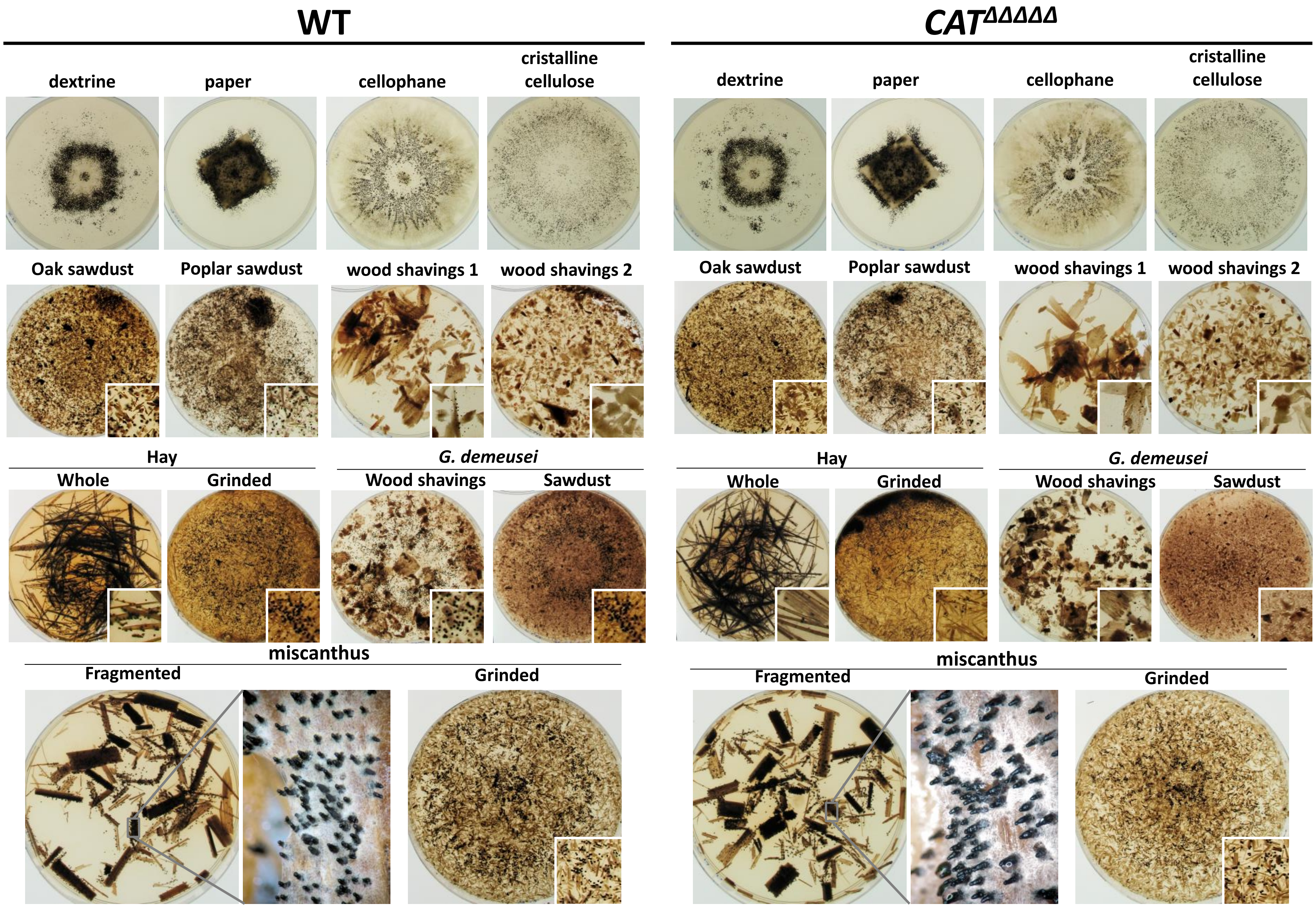

Figure S2. Fertility of *P. anserina* on various lignocelluloses. Ø=8 cm-Petri plates containing M2, M4 and 15 mL of jellified M0 medium supplemented with the indicated carbon sources were inoculated with similar amounts of *mat+/mat-* heterokaryotic mycelia from the P. anserina S (WT) and $CAT^{\Delta\Delta\Delta\Delta\Delta}$ strain and incubated for two weeks, at which time the pictures were taken. The fruiting bodies appear as small black dots (enlarged in the insets).
