# Supplementary material for: Lignin Degradation and Its Use in Signaling Development by the Coprophilous Ascomycete Podospora anserina
Source: J Fungi (Basel). 2020 Nov 11;6(4):278. doi: 10.3390/jof6040278 (PMC7712204; doi:10.3390/jof6040278)
Supplement: Supplementary file 1 [file jof-06-00278-s001.zip › jof-945128-supplementary/Figure S3.pdf]

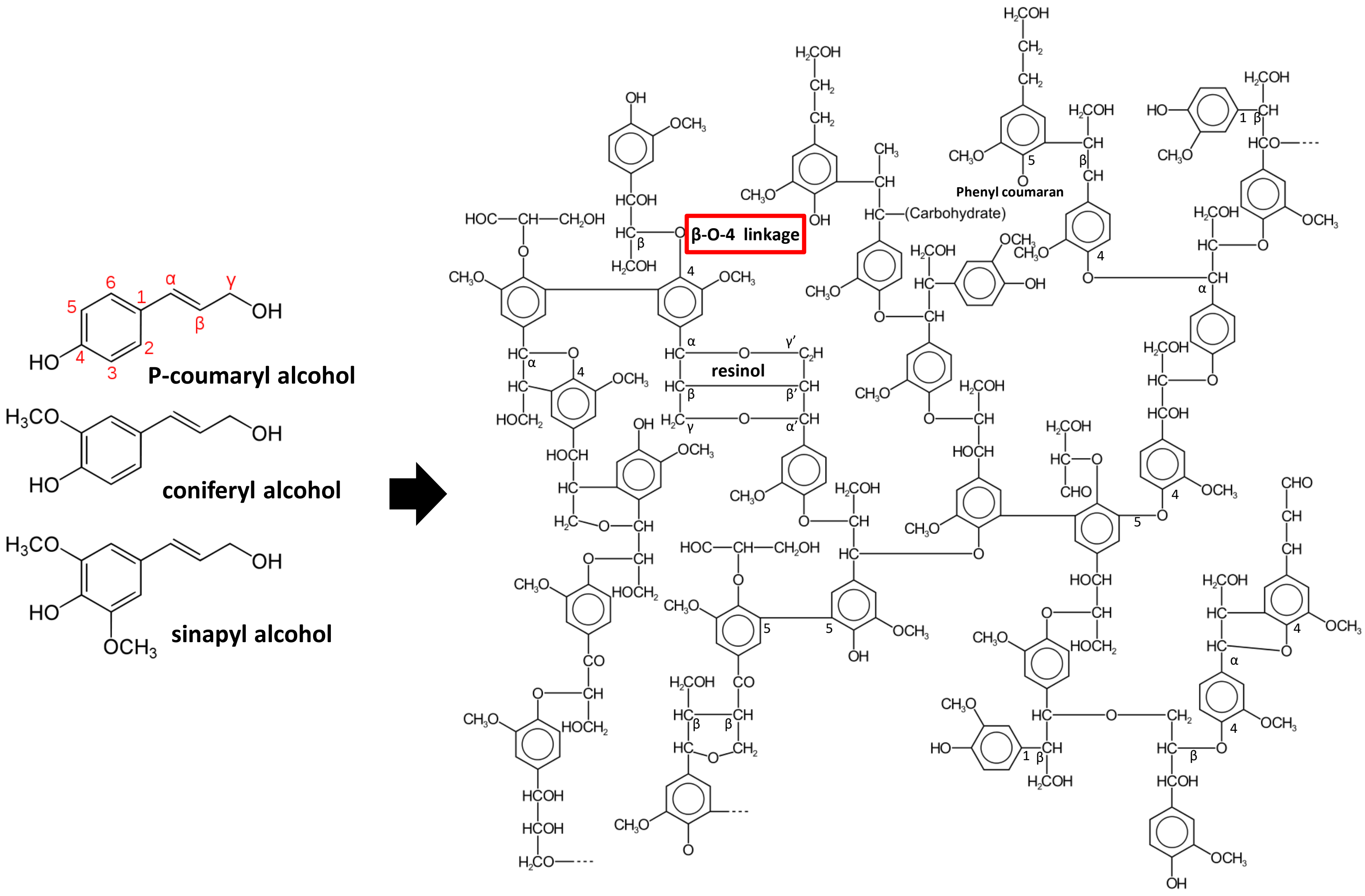

Figure S3. Lignin structure. Three building blocks of lignin or monolignols (coniferyl, sinapyl, and p-coumaryl alcohols; public domain image, courtesy of Chino) and hypothetic structure of lignin (adapted from public domain image). The different types of linkages are indicated and the main structures identified in MG lignin specified in bold characters. The β-O-4 linkage attacked by *P. anserina* is boxed.
