# Supplementary material for: Lignin Degradation and Its Use in Signaling Development by the Coprophilous Ascomycete Podospora anserina
Source: J Fungi (Basel). 2020 Nov 11;6(4):278. doi: 10.3390/jof6040278 (PMC7712204; doi:10.3390/jof6040278)
Supplement: Supplementary file 1 [file jof-06-00278-s001.zip › jof-945128-supplementary/Figure S4.pdf]

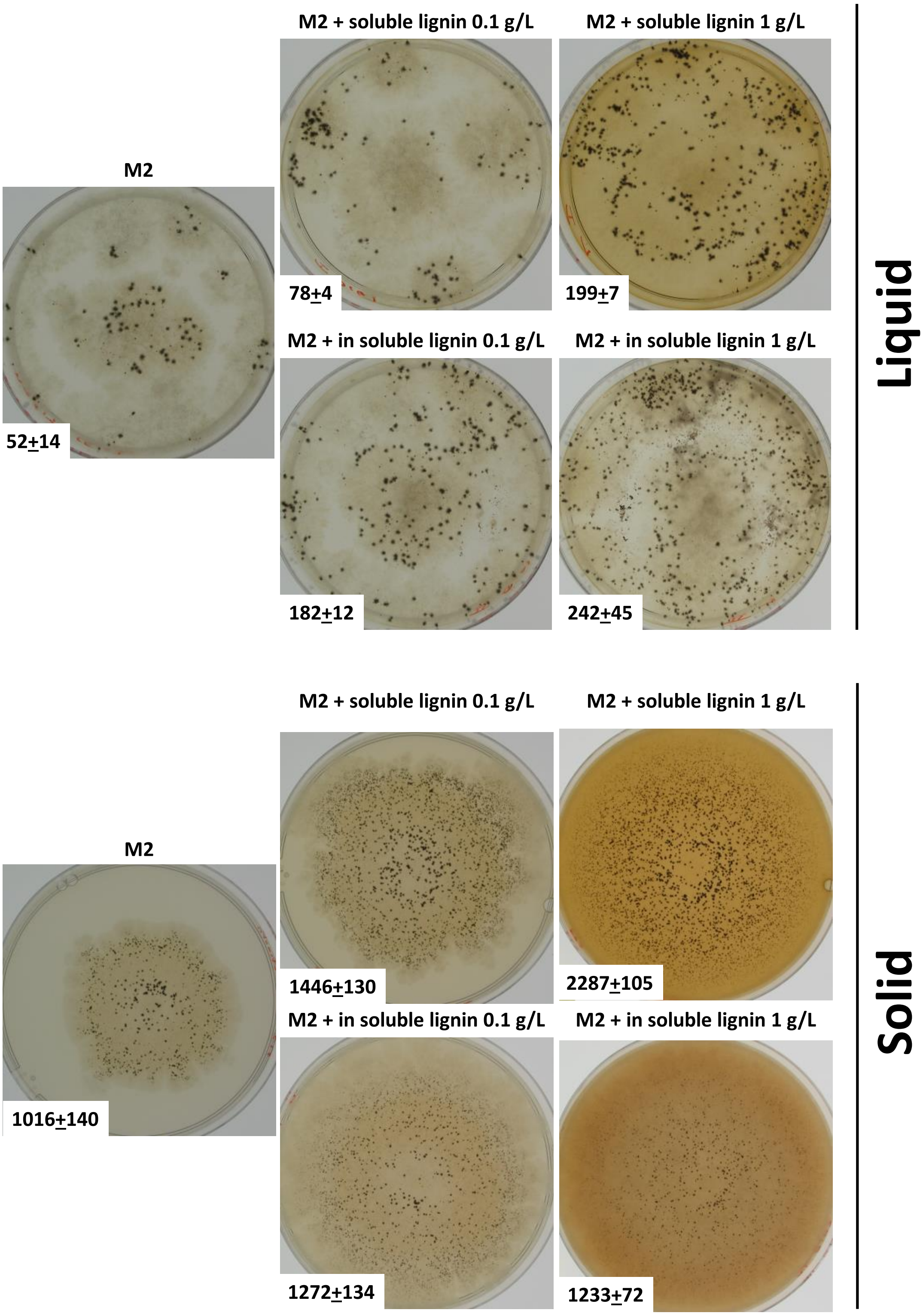

Figure S4: Fertility of *Chaetomium globosum* in the absence and presence of lignin. *C. globosum* was inoculated in Ø= 5 cm-Petri plates containing 5 mL of M2 medium supplemented or not with lignin (Top: Liquid) or in Ø= 9 cm-Petri plates containing 27 mL of jellified M2 medium supplemented or not with lignin (Bottom: Solid). Plates were incubated for 15 days at which time the pictures were taken. Fruiting bodies appear as black dots. Inset: owing to the light pigmentation of the mycelium, fruiting body numbers could easily be evaluated. The numbers are average +/- standard deviation of the number of perithecia recovered in three different plates. As seen on solid media, growth in the

presence of lignin is faster since the cultures have invaded the whole plates, unlike in the absence of lignin.
